# Cytotoxic and radiosensitising effects of a novel thioredoxin reductase inhibitor in breast cancer

**DOI:** 10.1007/s10637-021-01106-5

**Published:** 2021-03-25

**Authors:** Nurul A Abdullah, Martyn Inman, Christopher J. Moody, Sarah J Storr, Stewart G Martin

**Affiliations:** 1 Nottingham Breast Cancer Research Centre, School of Medicine, Biodiscovery Institute, University of Nottingham, University Park, Nottingham, NG7 2RD UK; 2 Present address: Biomedical Science Department, Faculty of Medicine and Health Sciences, Universiti Putra Malaysia, 43400 Serdang, Malaysia; 3 School of Chemistry, University of Nottingham, University Park, Nottingham, NG7 2RD UK

**Keywords:** Breast cancer, Radiosensitivity, Thioredoxin reductase, Indolequinone

## Abstract

Radiotherapy is an effective treatment modality for breast cancer but, unfortunately, not all patients respond fully with a significant number experiencing local recurrences. Overexpression of thioredoxin and thioredoxin reductase has been reported to cause multidrug and radiation resistance - their inhibition may therefore improve therapeutic efficacy. Novel indolequinone compounds have been shown, in pancreatic cancer models, to inhibit thioredoxin reductase activity and exhibit potent anticancer activity. The present study evaluates, using in vitro breast cancer models, the efficacy of a novel indolequinone compound (IQ9) as a single agent and in combination with ionising radiation using a variety of endpoint assays including cell proliferation, clonogenic survival, enzyme activity, and western blotting. Three triple-negative breast cancer (MDA-MB-231, MDA-MB-468, and MDA-MB-436) and two luminal (MCF-7 and T47D) breast cancer cell lines were used. Results show that treatment with IQ9 significantly inhibited thioredoxin reductase activity, and inhibited cell growth and colony formation of breast cancer cells with IC_50_ values in the low micromolar ranges. Enhanced radiosensitivity of triple-negative breast cancer cells was observed, with sensitiser enhancement ratios of 1.20–1.43, but with no evident radiosensitisation of luminal breast cancer cell lines. IQ9 upregulated protein expression of thioredoxin reductase in luminal but not in triple-negative breast cancer cells which may explain the observed differential radiosensitisation. This study provides important evidence of the roles of the thioredoxin system as an exploitable radiobiological target in breast cancer cells and highlights the potential therapeutic value of indolequinones as radiosensitisers.

***This study was not part of a clinical trial. Clinical trial registration number: N/A

## Background

Breast cancer is the most common form of cancer among women with an estimated 2 million new cases diagnosed across 185 countries in 2018 [1]. In the UK, an estimated 55,439 new cases and 11,849 deaths occurred from breast cancer in 2018 [1]. Treatment is often effective; however, according to the recent 2018 report by the Early Breast Cancer Trialists’ Collaborative Group, a significant proportion of early-stage breast cancer patients develop local recurrence (15.9% at 15 years) following adjuvant chemotherapy [2]. Radiotherapy is an important treatment option in the management of breast cancer [3], playing a key role in early-stage invasive, locally advanced, and metastatic breast cancers, either as curative or palliative treatments [3]. Although an effective cancer treatment modality, radioresistance may result in treatment failure [4]. There is a need to identify new agents that can be combined in a rational way, to make radiotherapy more effective. Cancer cells exist in conditions that result in elevated levels of reactive oxygen species (ROS) and, as a result, often develop highly effective antioxidant systems, with expression and/or function at higher levels than normal cells which may, in turn, promote tumour formation and progression [5]. A modest increase in ROS levels can speed up the rate of cancer cell proliferation by activating various signalling cascades linked to carcinogenesis such as the mitogen-activated protein kinase pathway. However, a further increase in ROS to a toxic level can activate ROS-induced cell death pathways including apoptosis, necrosis, and autophagy [6]. The upregulation of antioxidant systems may also protect cancer cells from the cytotoxic effect of certain therapies that rely upon induction of oxidative stress as a mechanism of action, both chemotherapeutic agents and ionising radiation. Therefore, modulating redox balance represent a potential strategy for cancer therapy.

The thioredoxin (Trx) system is an important antioxidant system involved in the maintenance of intracellular redox homeostasis and the radioresponse of cancer cells [7]. It is comprised of Trx, thioredoxin reductase (TrxR), NADPH, and the endogenous inhibitor of Trx; Trx-interacting protein (Txnip). TrxR plays a critical role in the oxidative stress process. It catalyses the reduction of oxidised Trx to its reduced and biologically active, state in the presence of NADPH [8]. Reduced Trx interacts with a number of biomolecules, reducing them in turn, including peroxiredoxins which are responsible for scavenging peroxides and protecting cells from an oxidative environment [9]. In cancer cells, Trx may exhibit different roles depending on the stage of cancer progression [10]. At the early stage of tumorigenesis, increased levels of Trx may assist in tumour development owing to its anti-apoptotic capabilities, however, as the cancer progress into a more advanced stage, Trx may promote cancer cell metastasis and angiogenesis [10–12]. Tumour cells often have high levels of Trx and TrxR than normal cells to cope with increased ROS demand and therefore are more vulnerable to inhibition of Trx/TrxR [13]. Previous studies have shown that the inhibition of TrxR activity elevates the formation of ROS which subsequently increases cancer cell sensitivity to irradiation [14, 15]. There is a growing interest in developing small molecule inhibitors of the Trx system, either as a single agent or used as adjuncts to existing anticancer agents. Many of these agents, however, have varying potency and target other thiols [13]. Hence, it is important to develop specific inhibitors that can only inhibit Trx or TrxR and not other enzymes.

**Fig. 1:**
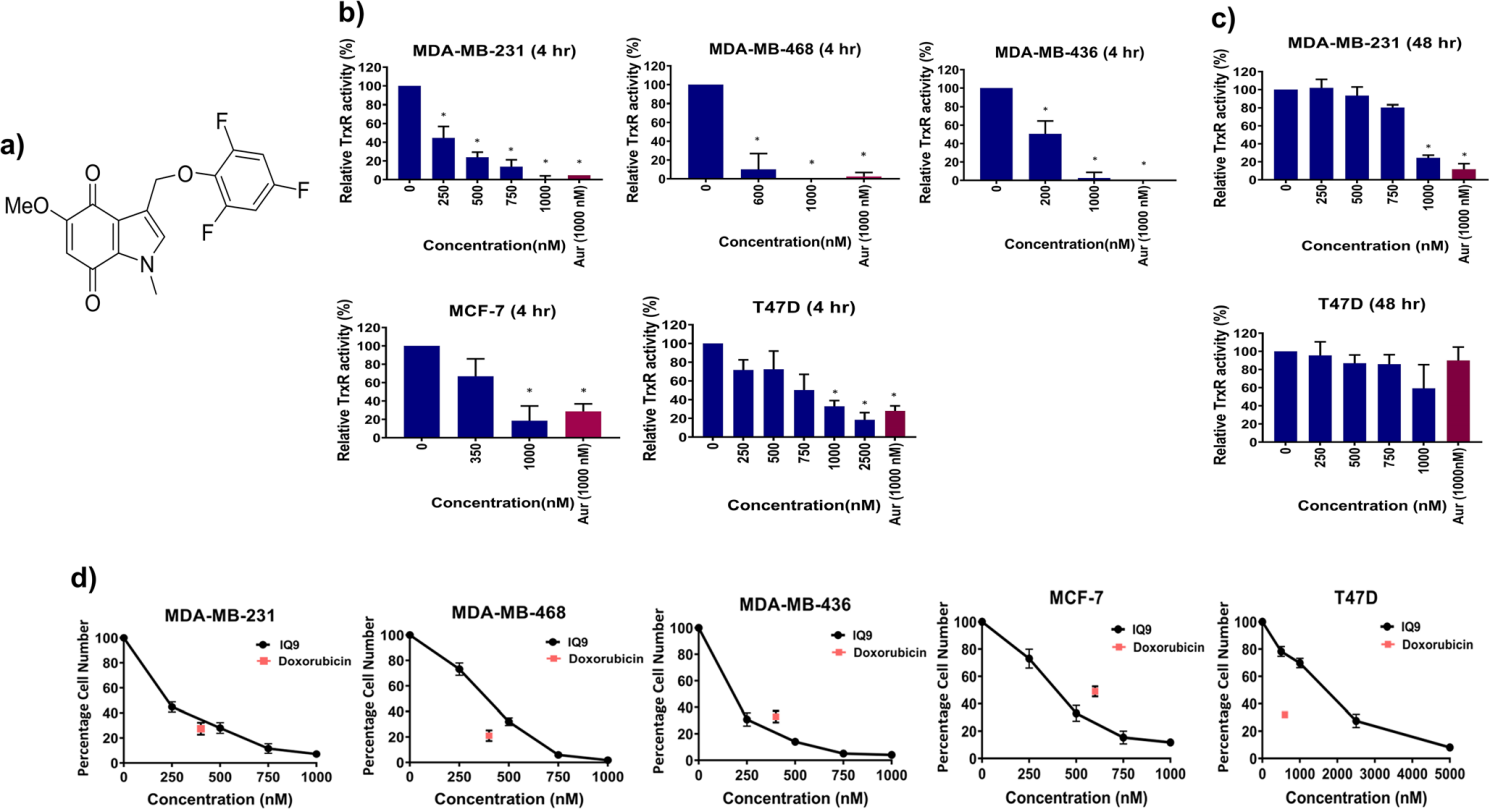
The inhibition properties of IQ9. **a** Chemical structure of IQ9. **b**,**c** TrxR activity after 4- or 48-h IQ9 treatment in breast cancer cell lines. Auranofin (1000 nM) was used as a positive control. Data represent the average TrxR activity ± SD of three independent experiments, with each experiment conducted in duplicate. **d** Effect of IQ9 on cell proliferation on breast cancer cells. Cells were treated with various concentrations of IQ9 (0–5000 nM) for 48 h. Doxorubicin was used as a positive control (400–600 nM). The average percentage of cell number (normalised to original seeding density) was plotted against IQ9 concentration. Data represent the average percentage cell number ± SD of three independent experiments, with each experiment performed in triplicate. *P < 0.05 vs control

Novel indolequinone derivatives (IQs), developed at the University of Nottingham, United Kingdom, have been previously reported as potent inhibitors of TrxR activity in pancreatic cancer cells and cell-free systems [16, 17], exhibiting potent anticancer activity in both in vitro and in vivo models [16, 17]. The activation of indolequinone agents requires two-electron reduction catalysis by reductases, loss of the leaving group, and the formation of iminium electrophiles that can alkylate TrxR at the C-terminal selenocysteine site. The covalent binding of quinone electrophiles to TrxR results in the irreversible inhibition of its activity [16]. The aims of the current study were to evaluate the potential therapeutic efficacy of one such indolequinone derivative, IQ9 (Fig. 1), as a single agent and in combination with radiation in breast cancer models. IQ9 was chosen as, based on structure-activity relationship (SAR) analysis, it is amongst the most potent of the IQs [17].

## Materials and methods

### Cell lines

Five human breast cancer cell lines were used, representing different breast cancer phenotypes: MDA-MB-231 and MDA-MB-468 cells (both triple-negative breast cancer phenotype (TNBC) with wild type BRCA-1) were maintained in minimal essential medium EAGLE (Sigma, UK) supplemented with 10% iron supplemented donor bovine serum (DBS) (Gibco, UK), 2 mM L-glutamine (Sigma, UK) and 1% penicillin /streptomycin (Sigma, UK). MDA-MB-436 cells (TNBC phenotype, with mutated BRCA-1) were maintained in Dulbecco’s Modified Eagle Medium/ Nutrient Mixture F-12 (Sigma, UK) supplemented with 10% iron supplemented DBS and 1% penicillin /streptomycin. MCF-7 and T47D (both luminal phenotypes) were maintained in RPMI1640 (Sigma, UK) supplemented with 10% iron supplemented DBS and 1% penicillin /streptomycin. All cell lines were originally obtained from the American Type Culture Collection and were cultured at 37 °C in a humidified incubator with 5% CO_2_. Cell lines were used within a 15-passage window. All cells were mycoplasma free and cancer cell line authentication was verified by short tandem repeat DNA profiling every 4–6 months (Promega Powerplex).

### Preparation of IQ9

IQ9 was synthesised at the School of Chemistry, University of Nottingham, United Kingdom by Professor Christopher Moody and Dr. Martyn Inman according to methods previously reported [16]. The purity of IQ9 was >95%.

### Thioredoxin reductase activity assay (insulin reduction)

TrxR enzyme activity was assessed using an insulin reduction assay [18]. Briefly, 2 × 10^6^ cells were seeded, in triplicate, in 75c m^2^ tissue culture flasks. After 24 h, sub-confluent cells were treated with either IQ9 (0–1000 nM) or 1000 nM auranofin (as a positive control) for 4 or 48 h, then trypsinised, washed, and resuspended in M-PER™ Mammalian Protein Extraction Reagent (ThermoFisher, USA) supplemented with 1X Halt protease inhibitor and EDTA. Protein concentration was determined using the Bradford assay. 80 μg of protein lysates isolated from each treatment conditions and reaction mix (HE buffer (100 mM HEPES pH 7.2, 5 mM EDTA), 20 μM Trx, 1.7 mM insulin, 10 mM β-NADPH) were added to the 96-well plate and incubated for 1 h at 37 °C. The reaction was stopped by adding stop buffer containing 6 M guanidine hydrochloride, 50 mM Tris, and 10 mM DTNB (Sigma, UK), with a final pH of 8.0. The plate was read at room temperature using a BMG Fluostar Optima Microplate Reader at 412 nM wavelength. The TrxR activity was expressed as the percentage of control, no drug-treated.

### Cell proliferation assay

1 × 10^5^ cells (2 × 10^5^ for T47D’s) were seeded, in triplicate, in 6-well plates and incubated overnight before being treated with IQ9 (0–5000 nM) or doxorubicin (400–600 nM) (positive control and comparator). Total cells were counted after 48 h using a haemocytometer. The total cell count in drug treatment wells was normalised as a percentage of the total cells in vehicle control wells.

### Clonogenic survival assay

5 × 10^5^ cells were seeded in T25 cm^2^ tissue culture flasks and incubated overnight. The sub-confluent cells were then treated either for 4-h or 48-h with IQ9 (0–5000 nM) or doxorubicin (10–20 nM). Following drug treatment, cells were collected, counted, and plated at low-density, in triplicate, and incubated, at 37 °C, 5% CO_2_, undisturbed, for 2 weeks for TNBC cells and 3 weeks for luminal cells, for colony formation. Colonies were fixed (50% methanol in 0.9% saline solution) and stained (0.5% crystal violet solution). Colonies consisting of more than 50 cells were confirmed by microscopy and scored as survivors. The plating efficiency (PE) was calculated as numbers of colonies formed/ numbers of cells plated. For single-agent treatment, drug or radiation, the surviving fraction was calculated as: number of colonies formed/ (numbers of cell plated x PE). For drug radiation combination experiments, cytotoxicity of drug treatment was accounted for by calculating surviving fraction as: number of colonies formed from each radiation dose/ (number of cells plated × PE × surviving fraction of drug-treated cell at 0 Gy).

### Cell irradiation

Sub-confluent cells were irradiated with a dose of 2, 4, 6, or 8 Gy (at a dose rate of 0.87 Gy/min) using an RS225 x-ray cabinet irradiation system (Xstrahl Limited, UK), fitted with a 0.5 mm Cu filter and run at 195 kV, 10 mA. Following irradiation, cells were immediately trypsinised and plated for clonogenic survival. Sham-irradiated cells were used as controls. Dose-response curves were plotted as a function of radiation dose on a log/ linear plot. Clonogenic survival calculation software (CS-CAL), developed by the Translational Radiation Oncology Group, German Cancer Research Centre was used to fit survival curves to the linear-quadratic (LQ) model (equation: $S={\mathit{\exp}}^{\left\lfloor -\left(\upalpha D+{\upbeta D}^2\right)\right\rfloor }$). The software can be accessed online at http://angiogenesis.dkfz.de/oncoexpress/software/cs-cal/. For drug-radiation combinations, cells were treated with 1000 nM IQ9 (2500 nM for T47D) for 4 h followed by irradiation (0–8 Gy). For 48 h drug treatment experiments, MDA-MB-231 and T47D cells were treated with clonogenic IC_50_ concentrations of IQ9 before irradiation. The sensitiser enhancement ratio (SER), calculated by dividing the X-ray dose causing 1% cell survival in the absence of drug treatment by the X-ray dose leading to 1% cell survival in the presence of drug treatment, was used to evaluate the degree of radiosensitisation.

### Western blotting

Cells were treated with IQ9 (clonogenic IC_50_ concentrations) for 48 h, harvested, and resuspended in 1 mL of RIPA buffer (Sigma, UK) supplemented with 1X Halt phosphatase inhibitor cocktail, protease inhibitor cocktail, and EDTA. Samples were run on an SDS-polyacrylamide gel and transferred onto a 0.2 μm nitrocellulose membrane (GE Healthcare). The membrane was then blocked using 5% milk powder in 0.1% PBS/ Tween20, for 1 h at room temperature and then incubated with primary antibody overnight at 4 °C. Anti-β-actin antibody (Abcam) was used as a loading control. Primary antibodies were rabbit anti-human Trx (Abcam; 1:1000), mouse anti-human antibody (Abcam; 1:500), and rabbit anti-human Txnip (Abcam; 1:500). For Trx and Txnip, bands were detected using an Odyssey FC Imager (LI-COR). Images were obtained and fluorescence intensity quantified using Image Studio Software (version 4). The signals for Trx and Txnip were normalised against β-actin. For TrxR, bands were detected using an Amersham Enhanced Chemiluminescence system (GE Healthcare).

### Statistical analysis

IC_50_ values were calculated from dose-response curves using an SPSS regression model between inhibition ratios and concentration gradients. The radiobiological parameters: alpha (α), beta (β), α/β ratio, and surviving fraction at 2 Gy (SF2) were extracted from the survival curves fitted using the LQ model. All results are presented as average ± standard deviation (SD) of three independent experiments, each performed in triplicate. Data were analysed using the student T-test and ANOVA one-way test. Statistical analysis was performed using SPSS 23.0 software. Values of *P* < 0.05 were considered statistically significant.

## Results

### IQ9 is an effective inhibitor of TrxR

IQ9 effectively inhibits TrxR activity more after 4-h than 48-h drug treatment, with inhibition being comparable to auranofin, and being more effective in TNBC than luminal cell lines (Fig. 1). Treatment with auranofin for 4 h, as the positive control, resulted in an approximately 70–100% decrease in TrxR activity across all cell lines. IQ9 at 1000 nM caused complete inhibition of TrxR activity in all TNBC cell lines however, in luminal cell lines approximately 70% inhibition was obtained. Treatment with auranofin for 48 h significantly inhibited approximately 80% TrxR activity in MDA-MB-231 (*P* < 0.001), however, in T47D cells, there was only 10% inhibition of TrxR activity compared to control. When the exposure time to IQ9 was increased to 48 h, a significant inhibition in TrxR activity was also observed in MDA-MB-231 cells, with no significant inhibition observed in T47D cells (Table 1).

**Table 1:** IC_50_ values for TrxR activity inhibition by IQ9

Cell lines	IC_50_ (nM)	
	4 h	48 h
MDA-MB-231	272.0 ± 144.6	759.2 ± 216.5
MDA-MB-468	439.5 ± 168.0	NA
MDA-MB-436	284.3 ± 169.8	NA
T47D	640.0 ± 250.4	1257.0 ± 660.0
MCF-7	619.0 ± 247.1	NA

Data represent the average ± SD of three independent experiments, with each experiment conducted in duplicate. *Abbreviations*: *NA* Not applicable

### IQ9 suppresses cell proliferation and inhibits colony formation of breast cancer cells

**Fig. 2:**
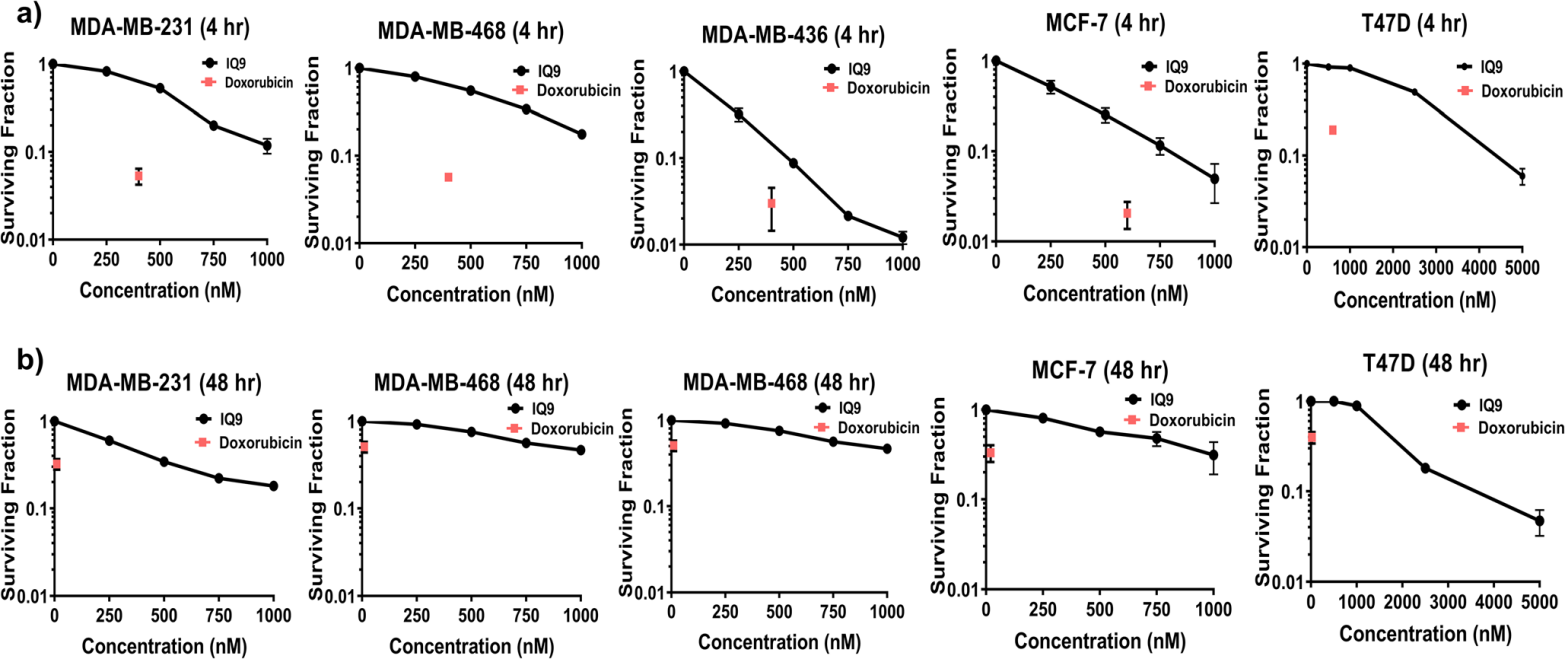
Effect of IQ9 on clonogenic survival of breast cancer cells. Cells were treated with various concentrations of IQ9 (0–5000 nM) for 4 or 48 h (**a**, **b**). Doxorubicin was used as a positive control (10–600 nM). The cell survival curve is presented by plotted the surviving fraction against various concentrations of IQ9. PEs were 48.0 ± 4.4% (MDA-MB-231), 46.3 ± 3.2% (MDA-MB-468), 12.3 ± 4.5% (MDA-MB-436), 15.0 ± 5.0% (T47D) and 26.7 ± 12.2% (MCF-7). Data represent the average surviving fraction ± SD of three independent experiments, with each experiment performed in triplicate

The cytotoxic effect of IQ9 was assessed by proliferation and clonogenic survival assays with doxorubicin being used as a positive control and drug comparator in each case. Cells behaved as expected, from the published literature, following doxorubicin treatment (400–600 nM), giving 50–70% decrease in cell number across all cell lines [19, 20]. In terms of antiproliferative effects, IQ9 behaved comparably to doxorubicin with only T47D’s being significantly more responsive to doxorubicin. Treatment with IQ9 for 48 h decreased breast cancer cell growth in a dose-dependent manner (Fig. 1) with a 90% decrease in cell number observed at the highest concentration used (1000 nM) across all cell lines. A dose-dependent decrease in clonogenic survival was also observed, at both 4- and 48-h drug treatments (Fig. 2) however, in this instance doxorubicin was substantially more potent. The 48-h proliferation IC_50s_ were, for IQ9, lower than the clonogenic IC_50_ across all cell lines (Table 2).

**Table 2:** Proliferation and clonogenic survival IC_50_ values

Cell lines	IC_50_ (nM)		
	Cell proliferation	Clonogenic survival	
	48 h	4 h	48 h
MDA-MB-231	246.5 ± 55.3	565.0 ± 29.5	324.3 ± 20.1
MDA-MB-468	425.3 ± 154.7	501.6 ± 170.7	916.1 ± 173.1
MDA-MB-436	171.3 ± 81.4	194.6 ± 4.5	235.8 ± 71.5
T47D	1432.3 ± 254.8	2072.0 ± 53.7	1880.0 ± 297.7
MCF-7	378.7 ± 42.4	340.5 ± 2.1	600.9 ± 113.6

Data represent the average ± SD of three independent experiments, with each experiment performed in triplicate

### IQ9 sensitises breast cancer cells to radiation following 4-h drug treatment

**Fig. 3:**
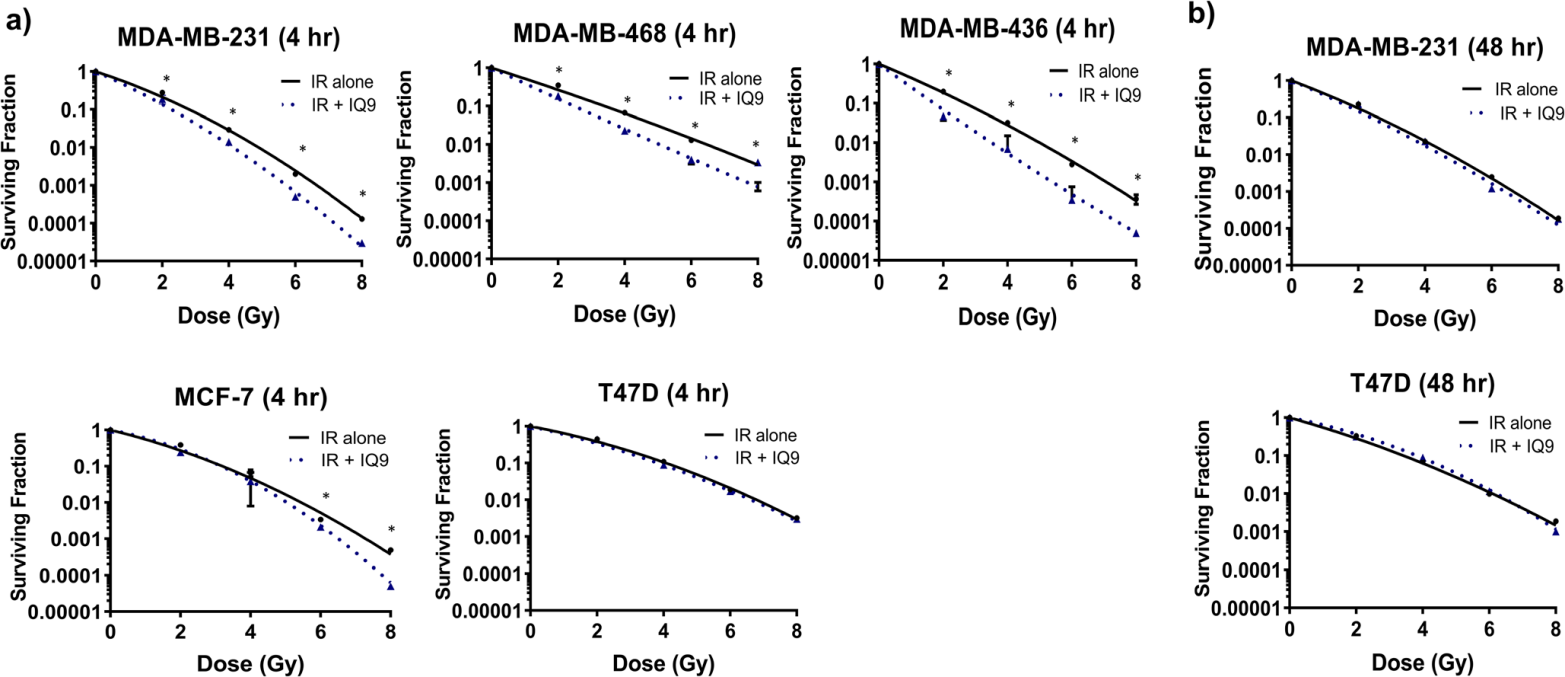
Effect of IQ9 on radiosensitivity of breast cancer cells after 4- and 48-h treatments (**a**, **b**). The radiation survival curves were fitted using the LQ model. PEs were 61.0 ± 10.0% (MDA-MB-231), 35.4 ± 2.7% (MDA-MB-468), 11.6 ± 1.1% (MDA-MB-436), 27.3 ± 1.3% (MCF-7) and 17.0 ± 4.3% (T47D). Data represent the average surviving fraction ± SD of three independent experiments with each experiment conducted in triplicate. (*P < 0.05 vs control)

As can be seen from Fig. 3, all TNBC cell lines showed a similar response to irradiation with SF2 values ranging from 0.19 to 0.36 and α/β ratio ranging from 10.28 to 30.03. The two luminal cell lines were slightly more resistant to irradiation with SF2 values from 0.38 to 0.47 and α/β ratio from 8.16 to 10.25. The radioresponse of the five breast cancer cell lines used in the current study was as expected and comparable to SF2 values in the literature [21, 22]. Treatment with IQ9 for 4 h significantly increased the radiosensitivity of MDA-MB-231, MDA-MB-468, and MDA-MB-436 cells to irradiation at 2, 4, 6, and 8 Gy (*P* < 0.05 for all doses) with SER values of 1.20, 1.30, and 1.43, respectively (radiobiological parameters are shown in Table 3). In MCF-7 cells, a significant increase in radiosensitivity was only observed above 6 Gy irradiation (SER value 1.08), however, no altered radiosensitisation was observed in T47D cells. When the drug exposure time was increased to 48 h, no significant increase in radiosensitivity was observed with either MDA-MB-231 or T47D cells (Fig. 3). Table 3 summarises the SERs, α, β, α/β ratio and SF2 values for all breast cancer cells.

**Table 3:** The effect of IQ9 on LQ parameters after 4- and 48-h drug treatment

Time	Cell lines	Treatment	SF2	α (Gy^−1^)	β (Gy^−2^)	α/β Ratio	SER
4 h	MDA-MB-231	Control	0.28 ± 0.01	0.61 ± 0.08	0.06 ± 0.01	10.28 ± 2.84	1.20
		IQ9	0.21 ± 0.02*	0.70 ± 0.17	0.06 ± 0.01	11.67 ± 3.63	
	MDA-MB-468	Control	0.36 ± 0.05	0.61 ± 0.04	0.02 ± 0.00	30.03 ± 3.62	1.30
		IQ9	0.19 ± 0.01*	0.85 ± 0.08*	0.01 ± 0.00	85.00 ± 8.18	
	MDA-MB-436	Control	0.19 ± 0.04	0.74 ± 0.11	0.03 ± 0.01	24.66 ± 12.93	1.43
		IQ9	0.07 ± 0.01*	1.33 ± 0.06*	0.02 ± 0.00	66.53 ± 2.89	
	T47D	Control	0.47 ± 0.03	0.41 ± 0.10	0.04 ± 0.01	10.25 ± 5.01	1.00
		IQ9	0.45 ± 0.02	0.47 ± 0.04	0.03 ± 0.00	15.66 ± 1.17	
	MCF-7	Control	0.38 ± 0.10	0.49 ± 0.06	0.06 ± 0.01	8.16 ± 1.50	1.08
		IQ9	0.24 ± 0.10	0.58 ± 0.18	0.08 ± 0.02	7.25 ± 3.19	
48 h	MDA-MB-231	Control	0.24 ± 0.04	0.71 ± 0.10	0.05 ± 0.01	13.31 ± 5.84	1.07
		IQ9	0.22 ± 0.08	0.85 ± 0.04	0.03 ± 0.01	25.60 ± 4.62	
	T47D	Control	0.33 ± 0.03	0.48 ± 0.13	0.05 ± 0.01	9.59 ± 5.59	1.00
		IQ9	0.31 ± 0.04	0.40 ± 0.12	0.05 ± 0.02	7.27 ± 5.53	

*Abbreviations*: *SF2* surviving fraction at 2 Gy. α represents the initial slope and β refers to the terminal slope of the dose-response curve. α/β ratio is the dose where the linear and quadratic components are equal. SER is the sensitiser enhancement ratio. (*P < 0.05 vs control). Data represent the average ± SD of three independent experiments with each experiment conducted in triplicates

### IQ9 differentially regulates the expression of Trx family proteins in breast cancer cells

**Fig. 4:**
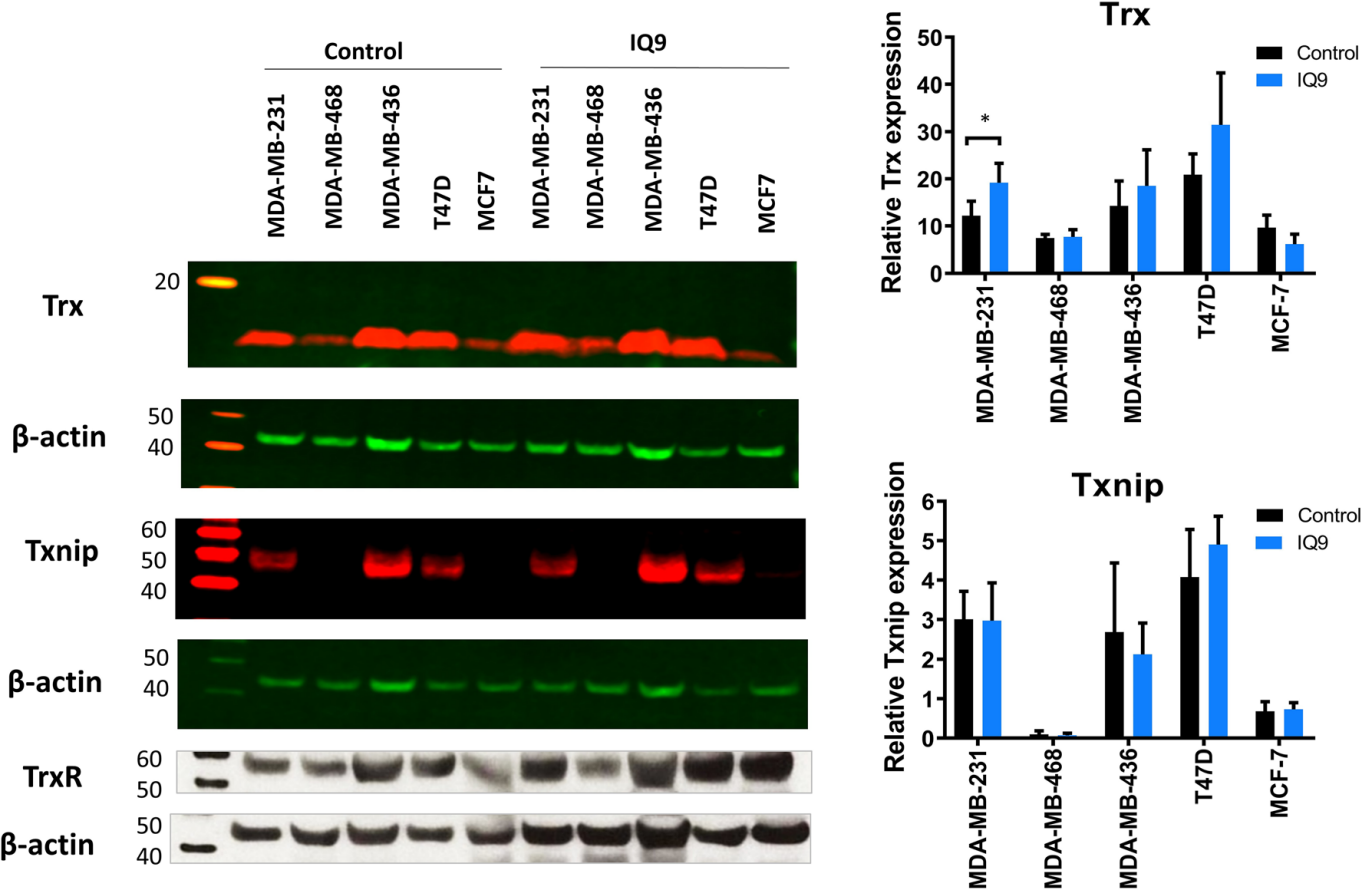
Effect of IQ9 on the expression of Trx family protein expression. Representative Western blots of three independent experiments. β-actin was used as the loading control (42 kDa). The signals of Trx and Txnip were normalised by β-actin signals. (*P < 0.05 vs control)

The effect of IQ9 on the expression of Trx system proteins (Trx, TrxR, and Txnip) was assessed across the five breast cancer cell lines by Western blotting (Fig. 4). T47D cells expressed the highest levels of Trx and Txnip compared with the other four cell lines. The endogenous expression level of TrxR was lower in MCF-7 than in MDA-MB-231, MDA-MB-468, MDA-MB-436 and T47D cells. IQ9 increased expression of Trx in MDA-MB-231 (*P* = 0.003) but had no effect on the other four cell lines. Increased expression of TrxR was seen in luminal cells following drug exposure but with little effect, if any, in TNBC cells. Txnip expression was not affected by IQ9 treatment in any of the breast cancer cell lines, with no pattern evident between phenotypes.

## Discussion

Deregulation of the Trx system has been observed in various diseases including cancer [23], stroke [24], and cardiovascular diseases [25]. Overexpression of TrxR protein has been reported in many solid tumours, leading to increased TrxR activity that may contribute to tumour development through its growth-promoting capacities [10]. Previous data have shown increased antioxidant abilities may make cancer cells less responsive to treatments that rely upon the generation of oxidative stress as their mechanism of action, such as radiotherapy [26, 27]. Anticancer drugs such as cisplatin [28] and motexafin gadolinium [29] have been reported to be TrxR inhibitors, suggesting that targeting TrxR may, therefore, be a useful therapeutic strategy both as a single modality and also to combine with radiotherapy.

Previous studies have shown that IQ derivatives are potent inhibitors of TrxR, however, no studies have been previously conducted to assess the cytotoxicity or radiosensitising effects of these agents in breast cancer models. Current TrxR enzyme activity data show that IQ9, at 4-h drug treatment, is more efficient in all TNBC than luminal breast cancer models. Also, IQ9 more effectively inhibits TrxR enzyme activity in MDA-MB-231 and T47D cells after 4-h than 48-h IQ9 treatment. Such findings support previous findings of TrxR being a target of indolequinones derivatives [16]. IQ9 gave comparable effects to auranofin, the positive control, and comparator. Auranofin is a well-characterised TrxR inhibitor and has entered phase I/II clinical trials against lymphoma (NCT01419691) and lung (NCT01737502) cancer.

The inhibition of TrxR activity has been shown to decrease cell proliferation and cell survival of cancer cells [30]. In the current study, IQ9 demonstrated a potent anticancer effect with the ability to inhibit cell growth and colony formation of all five breast cancer cells. When compared against the positive control drug, doxorubicin was more potent than IQ9 when measured by clonogenicity. The mechanism of action by which doxorubicin acts upon cancer cells is via intercalation between DNA base pairs on double helix and disruption of topoisomerase-II-mediated DNA repair [31]. Any disturbance in DNA damage pathways may lead to significant loss of clonogenic survival of cancer cells [32]. In comparison to other TrxR inhibitors, IQ9 appears to be more potent against breast cancer cells with IC_50_ ranges from 0.2 to 2 μM compared to auranofin [33] (IC_50_ between 2 and 10 μM) [33]. Data suggest that IQ9 is a potentially effective anticancer candidate for breast cancer. The cytotoxic effect of IQ9 on breast cancer cells may be linked with the inhibition of TrxR activity. A previous study reported that indolequinone derivatives were effective at inhibiting TrxR activity and cell growth of pancreatic cancer cell lines with IC_50’s_ in the low nanomolar range [16]. The current study also demonstrates that IQ9 inhibits breast cell growth, however, slightly higher concentrations were required suggesting that indolequinone derivatives have different potencies in different cancer cell types. In pancreatic cancer cell lines, the inhibition of TrxR by indolequinones caused a shift in the redox state and activated p38/c-Jun NH2-terminal kinase which subsequently led to the induction apoptosis [17]. Another study reported that knockdown of TrxR1 decreased cell proliferation and colony growth of multiple myeloma [34], whereas, in an animal study, TrxR-1 knockout led to embryonic lethality in mice [35].

Preclinical studies have shown that modulation of redox homeostasis could alter the response of cancer cells to low LET radiations often used in conventional radiotherapy [7, 36]. Current data demonstrate IQ9 treatment for 4 h followed by irradiation resulted in significant increases in radiosensitivity of TNBC cells with SER values ranging from 1.20 to 1.43 but with little effect in luminal breast cancer models. Such data suggest that IQ9 regulates breast cancer radiosensitivity in a phenotypic-specific manner. In terms of radiobiological parameters, treatment with IQ9 significantly increased α in MDA-MB-436 and MDA-MB-468 by a factor of 1.8 and 1.4, respectively, which represents a beneficial radiosensitisation, and the α/β ratio becomes so large that the resulting survival curves are effectively a straight line (rather like the case when high LET radiations are used). This suggests that there may be no fractionation benefit in these cell lines when the drug is added, and the combined cell kill and enhanced radiosensitivity would additionally allow the total dose to be reduced. When breast cancer cells were treated with IQ9 for 48 h followed by irradiation no radiosensitisation was observed in either TNBC or luminal phenotypes - the lack of TrxR enzyme inhibition at 48-h IQ9 treatment may explain such lack of radiosensitisation at this time point. Inhibition of TrxR activity has been shown to increase response to radiotherapy [36, 37]. The level of radiosensitisation of IQ9 observed in this study is comparable with curcumin. In previous preclinical studies, curcumin has been shown to improve radiosensitivity of renal (SER 1.42) [38] and breast (SER 1.38–1.78) cancer lines [39].

MDA-MB-436, a BRCA1 deficient cell line, was shown to be the most sensitive to IQ9 treatment with the lowest IC_50_ value in cytotoxicity and enzyme activity assays, and greatest SER than those cell lines with functional BRCA1 status. BRCA1 is a protein that plays a major role in DNA repair [40]. Earlier studies have shown that cells carrying a mutation of BRCA genes display lower clonogenic survival [41, 42]. In this study, T47D cells were the most resistant to IQ9, radiation alone, and IQ9-radiation combination treatments. The high level of endogenous Trx in T47D cells may be one of the factors contributing to the resistance of this cell line in both cytotoxic and radiation combination experiments. High expression of Trx is associated with resistance to several chemotherapeutic agents such as docetaxel [43].

Treatment with IQ9 significantly increased the expression of Trx in MDA-MB-231 cells, but this was not observed in other cell lines suggesting that IQ9 may regulate Trx expression, although the effect may be cell type-specific. IQ9 upregulated the expression of TrxR in luminal but not in TNBC cells suggesting that luminal cells may increase TrxR expression to compensate the inhibition in TrxR activity. Inhibition of TrxR, and ultimately the entire Trx system, contributes to the induction of oxidative stress [44]. Treatment with IQ9 may induce oxidative stress conditions inside the cells, activating signalling pathways that regulate antioxidant enzyme expression. The observed changes in expression of the enzymes may indicate the expression required in individual cell lines to attempt to maintain an intracellular redox balance. In glioma cells, treatment with novel TrxR1 inhibitors increased mRNA expression of Trx and TrxR1 in response to high ROS levels [45]. In addition, an increase in the TrxR expression may be one of the reasons why luminal cells did not show as much increased radiosensitivity following IQ9 treatment. A recent study demonstrated that radiosensitivity of glioma cells was decreased by TrxR1 overexpression [46].

The present data demonstrate that the efficacy of IQ9 decreases following longer exposure. IQ9 is an analogue of ES936, an NQO1 inhibitor developed from EO9 which has been shown to be a potent anticancer agent against pancreatic cancer cells [47]. Based on the biostability study of ES936, the ability to inhibit NAD(P)H: quinone oxidoreductase 1 activity was only observed between 2 to 4 h of incubation in complete media [48]. On the other hand, EO9, a synthetic derivative of mitomycin C has been shown to only penetrate a few microns from blood vessels and has rapid clearance [49]. Taken together, these indicate that indolequinone compounds are only active under short-term exposure. When a drug has a short half-life, frequent dosing may be required to maintain the desired effects; however, this may pose a challenge to achieve optimal efficacy and minimised toxicity [50]. Clinically many chemotherapy drugs, such as 5-fluorouracil (5-FU), have relatively short half-lives (less than 20 min for 5-FU) and are administered as continuous intravenous infusions [51].

In summary, IQ9 is a novel anticancer agent with the ability to inhibit breast cancer cell growth and survival at low micromolar concentrations. It preferentially sensitises TNBC to ionising radiation if irradiated shortly after drug exposure. The increase in the radiosensitivity by IQ9 may be due to the inhibition of TrxR activity, suggesting that modulating the Trx system may alter radioresponse. Additional work examining radiation fractionation, altered dosing regimens, and incorporating in vivo animal models is warranted to determine the safe dose and to assess for any potential toxicities.

## Abbreviations

BRCA1: Breast cancer gene 1
DBS: Donor bovine serum
LQ: Linear quadratic
PE: Plating efficiency
ROS: Reactive oxygen species
SER: Sensitiser enhancement ratio
SF2: Surviving fraction at 2 Gy
TNBC: Triple-negative breast cancer
Trx: Thioredoxin
TrxR: Thioredoxin reductase
Txnip: Thioredoxin-interacting protein
